# Compressed autograft biceps tendon augmentation of subscapularis repair following shoulder arthroplasty

**DOI:** 10.1016/j.xrrt.2022.08.002

**Published:** 2022-08-24

**Authors:** Patrick J. Denard, Javier Ardebol, Ignacio Pasqualini, Jeffrey L. Horinek, Joshua Dines, John M. Tokish

**Affiliations:** a Oregon Shoulder Institute, Medford, OR, USA; b Hospital for Special Surgery, New York, NY, USA; c Mayo Clinic, Scottsdale, AZ, USA

**Keywords:** Shoulder, Arthroplasty, Autograft, Biceps, Subscapularis, Augmentation

## Abstract

Subscapularis integrity is correlated with function following shoulder arthroplasty. Failure of healing, particularly following anatomic total shoulder arthroplasty, is associated with poor outcomes and the need for revision. Graft augmentation has been used to increase healing following rotator cuff repair but has not been commonly advocated for augmenting the subscapularis following shoulder arthroplasty. The long head biceps tendon is typically tenodesed and discarded during shoulder arthroplasty. Rather than discarding the tendon, the tendon may be compressed and applied to the subscapularis as a biologic scaffold to potentially improve subscapularis healing following shoulder arthroplasty.

Subscapularis healing following shoulder arthroplasty is crucial for maintaining glenohumeral joint stability and optimizing functional outcomes.^2^ Lack of subscapularis healing has been associated with pain, instability, decreased range of motion, and possibly revision surgery.^14^ Despite advances in tendon repair techniques, healing rates after total shoulder arthroplasty (TSA) and reverse shoulder arthroplasty (RSA) range from 76% to 93% and 40% to 53%, respectively.^1^^,^^7^^,^^9^ Thus, there is continued room for improvement.

Biologic augmentation of arthroscopic rotator cuff repairs, most commonly with humeral dermal allograft or dermal xenograft, has been reported to improve biomechanical strength and tendon healing.^16^ Others have proposed using the long head biceps tendon (LHBT) to augment cuff repairs.^6^^,^^18^^,^^19^ Recently it has been demonstrated that compressed LHBT retains tenocyte viability, suggesting that this graft source has biologic potential to facilitate healing.^8^ This autograft poses several advantages including low cost, reduced risk for adverse reactions, and decreased graft site morbidity. Despite the importance of subscapularis healing, there has been very little discussion of augmenting subscapularis repair following shoulder arthroplasty. During shoulder arthroplasty, the LHBT is routinely tenodesed or tenotomized, followed by discarding the proximal segment. In lieu of discarding this portion, there is potential to compress the proximal segment of the LHBT and apply it to the subscapularis repair as an autologous biologic augmentation. The purpose of this technical note is, therefore, to describe a method for preparing and applying an autologous compressed LHBT graft to the subscapularis repair during shoulder arthroplasty.

## Technique

### Indications

The only requirement for considering LHBT augmentation of the subscapularis repair is the presence of the proximal attachment of the LHBT. The presence of fraying or synovitis of the LHBT encountered intraoperatively does not necessarily exclude the patient for biological augmentation.^8^

### Surgical technique

The steps of the procedure are displayed in Table I and Video 1. The patient is placed in a beach chair position followed by a standard deltopectoral approach. The LHBT is located at the superior aspect of the pectoralis major. The tendon is first tenodesed to the pectoralis major tendon with 2 #2 sutures. The tendon is then cut just above the tenodesis site, the bicipital groove is opened, and the proximal segment of the tendon is resected. A segment of at least 30 mm is harvested, although the typical length available is 50 to 60 mm. The harvested tendon is transferred to the back table and then wrapped in a saline-soaked gauze. The subscapularis is taken down based on surgeon preference, followed by the conclusion of the shoulder arthroplasty.

**Table I:** Steps for compressed biceps tendon augmentation of subscapularis repair following shoulder arthroplasty.

• Tenodese the biceps tendon to the upper border of the pectoralis major tendon
• Harvest the proximal segment of the long head biceps tendon and place in saline solution (0.9%)
• Proceed with subscapularis takedown to access the glenohumeral joint and complete shoulder arthroplasty
• Measure and cut a 27 mm long section of the biceps tendon
• Compress the biceps tendon in trays for 4 minutes
• Sew the compressed biceps graft into the native subscapularis tendon

### Graft preparation

**Figure 1:**
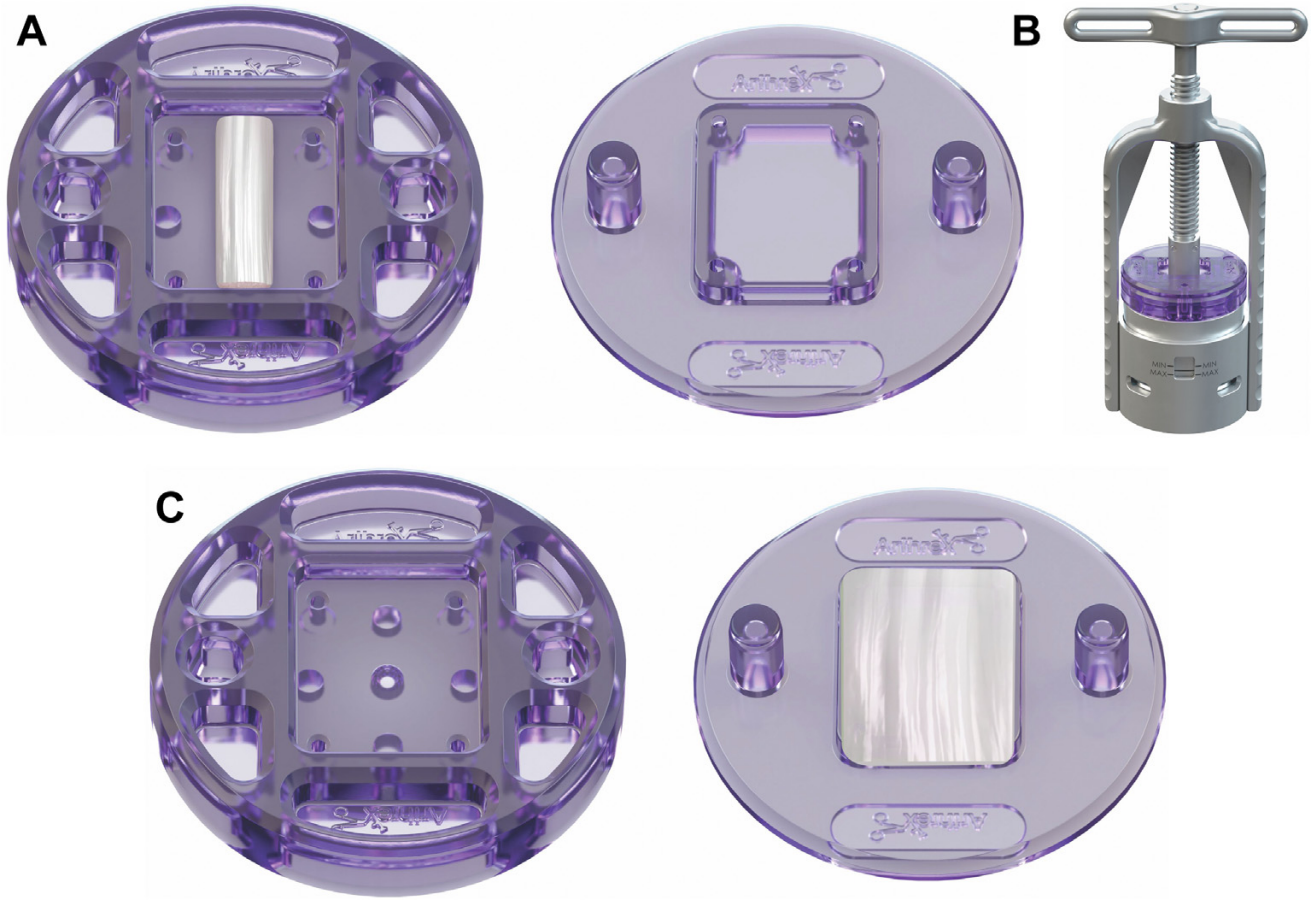
Illustration of the LHBT preparation. (**A**) A 27 mm long segment of the LHBT is centered in the compression tray (Biceps Compression Tray; Arthrex Inc.). (**B**) The trays are placed into the device while holding compression between the minimum and maximum values (Modular Glenoid System; Arthrex Inc.). (**C**) Compressed biceps patch. *LHBT*, long head of the biceps tendon.

Prior to performing the subscapularis repair, the compressed biceps graft is prepared on the back table. If the level of the proximal resection was flush with the labrum, the proximal 5 mm of the graft is discarded as this segment tends to be more cartilaginous and not compress as well. A 27 mm segment from the proximal aspect is then obtained (Fig. 1). The tendon is placed in a prefabricated tray (Biceps Compression Tray; Arthrex Inc., Naples, FL, USA). The tray consists of two plates which allow for compression of the biceps tendon into a 25 mm wide by 27 mm long graft. The trays are placed in a compression device (Modular Glenoid System; Arthrex Inc.). Compression is applied to between 2500 N and 3600 N and held for 4 minutes. Subscapularis repair is performed while compression is being applied to the biceps graft. The tray is then removed from compression after 4 minutes, and the graft is left in the tray until the subscapularis repair is completed. Prior to removing the graft from the tray, it is an option to pass sutures through preplaced holes in the tray to create mattress configurations at the 4 corners. Finally, given the length of the proximal tendon, it is possible to obtain 2 grafts if desired. These grafts can either be stacked in the subsequent augmentation, or placed side by side.

In the setting of a tenotomy approach, a side-to-side repair is performed in the standard fashion. The graft is then held over the tenotomy repair site with the biceps graft aligned from superior to inferior. This orientation makes it so that the sutures will pass perpendicular to the normal orientation of the biceps tendon fibers. The 4 corners of the graft are sutured to the native tendon using 4 independent #2 sutures placed in a figure-of-eight configuration.

**Figure 2:**
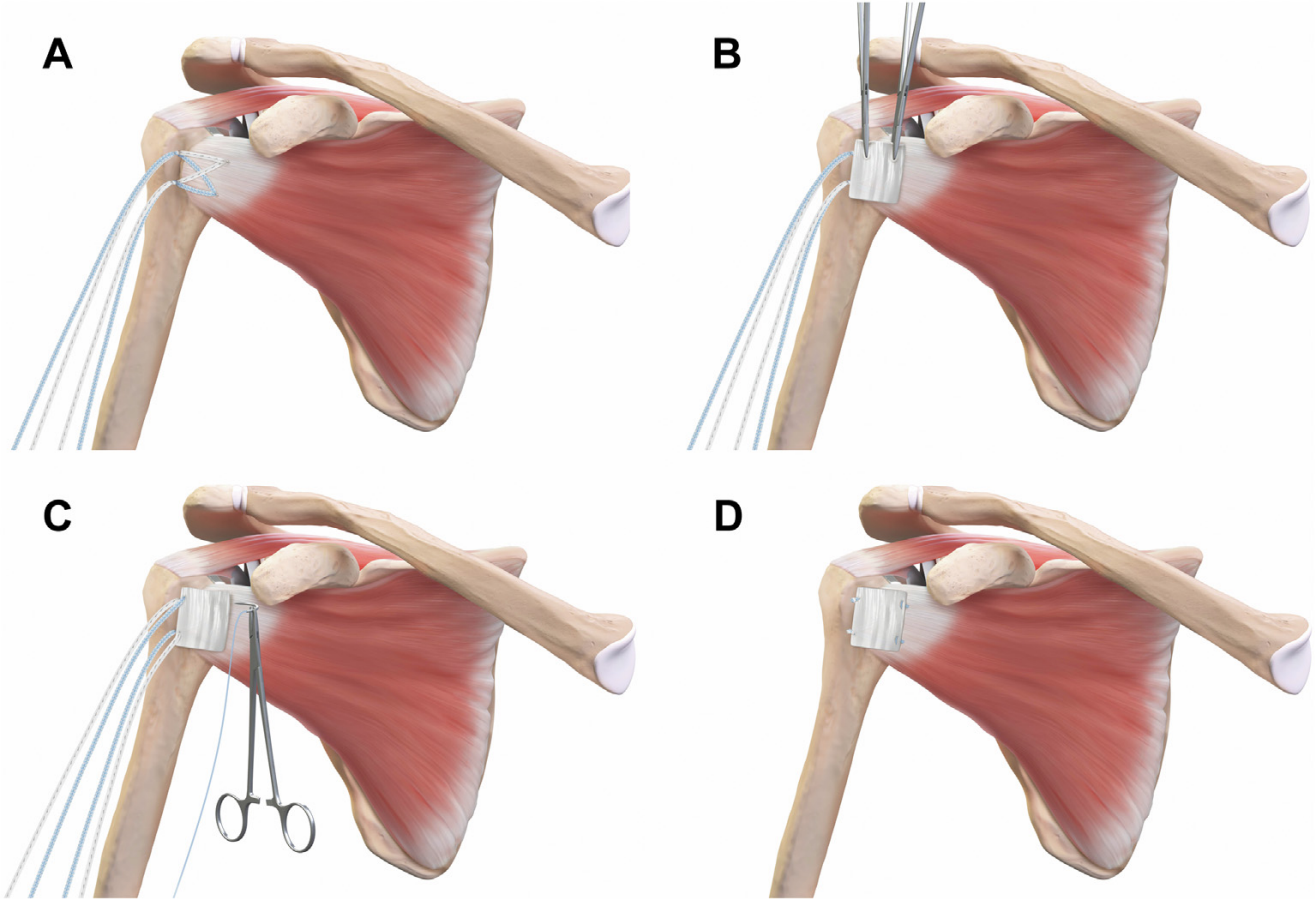
Illustration of repair of the subscapularis peel and overlay of the compressed biceps patch. (**A**) A tensionable construct is created to repair the subscapularis tendon. (**B**) The compressed biceps patch is laid over the repair construct. (**C**) The patch is secured laterally with suture limbs from the subscapularis repair, and medially with independent sutures in the medial tendon. (**D**) Sutured patch on all four corners.

Most commonly, we utilize a subscapularis peel or lesser tuberosity osteotomy approach and repair the tendon with 2 mm suture tapes (Tendon Compression Bridge; Arthrex, Inc.) (Fig. 2). The full technique has previously been described in detail.^10^^,^^11^ This repair results in a criss-crossed suture-bridging configuration with the lateral suture limbs secured in 2 half-racking hitches. Typically, the lateral limbs are cut at the completion of the repair, but in this setting, they are preserved. The graft is secured to the medial aspect of the native tendon with 2 sets of #2 sutures at the superomedial and inferomedial corners as described following tenotomy. Then, the lateral suture tape limbs are used to secure the graft laterally. One of the superior limbs is passed through the graft with a free needle and tied to the other superior limb. This is then repeated inferiorly to complete the augmentation.

## Discussion

The compressed biceps autograft augmentation technique provides a technique for potentially improving subscapularis healing following shoulder arthroplasty. The LHBT graft source is low cost, present in most shoulder arthroplasties, and has demonstrated cell viability following compression.

Subscapularis failure following TSA is a recognized cause of decreased function and implant failure.^3^^,^^13^ One study reported subscapularis insufficiency in 78% of failed TSAs.^17^ While a wide variety of techniques have been reported to attempt to improve the strength of repair, failures continue to occur. In a systematic review, Choate et al reported that subscapularis healing in the setting of TSA was 76% for tenotomy, 84% for peel, and 93% for lesser tuberosity osteotomy.^7^ Thus, particularly in the setting of a tenotomy or peel approach, there is a need for improvement. Much as the biomechanics of arthroscopic rotator cuff repair have been relatively optimized and attention has turned to biology, there may be a similar opportunity to improve subscapularis healing following TSA by optimizing the biologic environment.

The importance of subscapularis repair and healing following RSA has been a subject of controversy. Some studies have reported minimal to no difference in patient function whether or not the subscapularis was repaired following RSA.^15^ Other studies have associated increased likelihood of postoperative dislocation with irreparable subscapularis tendons.^4^^,^^5^^,^^12^ One reason for this debate may be the variable or underreported subscapularis healing rates in the setting of RSA. In the few studies that have evaluated subscapularis healing following RSA, the rate of healing has varied between 40% and 53%.^1^^,^^9^ Recently, Collin et al demonstrated that subscapularis healing was associated with improved internal rotation following RSA.^9^ They reported a significantly higher Constant IR score in the intact repair group compared to the nonrepair group (6.6 vs. 4.8 points, *P* = .0058). Thus, the lack of clarity regarding subscapularis repair following RSA may be partially due to the inherently low healing rates. Low healing rates in the setting of RSA can be attributed to distalization of the subscapularis tendon as well as the poor native biologic environment in the case of rotator cuff arthropathy. In the latter case, there may be an opportunity to improve healing and subsequent function with biologic augmentation.

The LHBT is a promising graft source for augmentation of subscapularis repair for several reasons. First, the graft source is low cost, making use of a segment of the tendon that is routinely discarded. Second, there is no donor site morbidity. Third, the graft appears to retain biologic viability even after compression. Colbath et al analyzed the biological and tensile properties of a compressed LHBT graft and demonstrated that 39% of tenocytes were viable.^8^ Additionally, they observed that the compressed cell produced cytokines that promote tenogenic differentiation in adipose-derived mesenchymal stromal cells. The findings suggest a biologic potential for augmentation of rotator cuff repair.

Further studies are clearly required to investigate this technique. First, a biomechanical investigation would be valuable to determine if the augmentation improves load to failure or minimizes displacement. Most importantly, a clinical study evaluating healing would be valuable. However, as opposed to allografts which require substantially increased cost, there appears to be very little downside to the current technique.

## Conclusion

During shoulder arthroplasty, the proximal segment of the long head of the biceps tendon is routinely discarded. Rather than discarding the tendon, we have described a technique for compressing the biceps into a flat graft for autologous biologic augmentation of subscapularis repair following shoulder arthroplasty.

## Disclaimers:

Funding: No funding was disclosed by the authors.

Conflicts of interest: Dr. Denard is a consultant for and receives royalties from Arthrex, Inc. Dr. Dines is a consultant for and receives royalties from Arthrex, Inc. Dr. Tokish is a consultant and receives royalties from Arthrex. He has recently served on the Scientific Advisory Board for Depuy Mitek, and is an Associate Editor for the Journal of Shoulder and Elbow Surgery. The other authors, their immediate families, and any research foundation with which they are affiliated have not received any financial payments or other benefits from any commercial entity related to the subject of this article.

## Supplementary Data

Video 1 The technique for prepairing and applying a compressed biceps tendon graft to augment subscapularis repair following shoulder arthroplasty.
